# Correlation of breast cancer risk factors with HER-2/neu protein overexpression according to menopausal and estrogen receptor status

**DOI:** 10.1186/1472-6874-5-1

**Published:** 2005-02-04

**Authors:** Nikos Tsakountakis, Elias Sanidas, Efstathios Stathopoulos, Maria Kafousi, Nektaria Anogiannaki, Vasilis Georgoulias, Dimitris D Tsiftsis

**Affiliations:** 1 Dept of Family and Social Medicine, Heraklion Medical School, University of Crete, Greece; 2 Dept of Surgical Oncology, Heraklion Medical School, University of Crete, Greece; 3 Dept of Pathology, Heraklion Medical School, University of Crete, Greece; 4 Dept of Biostatistics, Heraklion Medical School, University of Crete, Greece; 5 Dept of Medical Oncology, Heraklion Medical School, University of ≥Crete, Greece

## Abstract

**Background:**

Several researchers have claimed that classification of tumours on the basis of HER-2/*neu *overexpression or amplification may define a subset of breast cancer in which the net effect of a risk factor could be rather more obvious and its impact on breast cancer development more clear. We decided to investigate, in a group of patients from a geographical area with a low incidence of breast cancer, whether HER-2/*neu *positive tumours are correlated with established or suspected risk factors for breast cancer and thus to identify distinct subgroups of high risk women.

**Methods:**

This study analysed data from patients who attended the Breast Unit at the University Hospital of Heraklion, Crete, Greece between 1996 and 2002. 384 women with primary invasive breast cancer were compared with 566 screened women who were referred to the Unit and had not developed breast neoplasm by the time the data were analysed. Risk factor data were obtained from each subject by personal interviews using a structured questionnaire. The detection and scoring of the HER-2/*neu *protein, estrogen and progesterone receptor expression were performed using immunochemistry. Odds ratios and 95% confidence intervals were determined by chi-square test and logistic regression analysis. Case-case odds ratios were calculated in order to measure the risk heterogeneity between HER-2/*neu*+ and HER-2/*neu*-tumours. Separate analyses were performed for premenopausal and postmenopausal women and according to estrogen receptor status.

**Results:**

In multivariate analysis without HER-2/*neu *stratification, an increased breast cancer risk was associated with only *four *of the factors examined: use of oral contraceptives (OR = 4.40, 95%C.I: 1.46–13.28), use of HRT (OR = 7.34, 95%C.I: 2.03–26.53), an age at first full pregnancy more than 23 years (OR = 1.91, 95%C.I: 1.29–2.83) and body mass index more than 29 kg/m^2 ^(OR = 3.13, 95%C.I: 2.02–4.84). Additionally, a history of abortion or miscarriage (OR = 0.56, 95%C.I: 0.38–0.82) was correlated with a decreased risk of breast cancer. In the case to case comparison only BMI >29 kg/m^2 ^revealed a relative connection that was stronger with positive than with negative HER-2/*neu *tumours (ratio of OR's = 2.23, 95%C.I: 1.20–4.15, p = 0.011). This may indicate evidence of heterogeneity of a rather significant degree for this factor. In the ER negative group an age at first full pregnancy >23 years and a BMI >29 kg/m^2 ^were associated with an increased risk in both HER-2/*neu *groups, but the association was significantly stronger for the latter factor in the positive HER-2/*neu *tumours (ratio of OR's = 2.46, 95%CI: 0.97–6.21).

**Conclusions:**

Our study did not confirm that the established or putative hormonal breast cancer risk factors differ regarding their relations with HER-2/*neu*+ versus HER-2/*neu*-breast tumours, with the exception of increased BMI. Further innovative studies with larger sample sizes are needed to examine how the status of these potentially modifiable breast cancer risk factors interacts with biological markers such as HER-2/*neu *oncoprotein.

## Background

The HER-2/*neu *oncogene, also known as c-erb-B2, c-*neu *or ERBB2, is located in chromosome 17q11.2-12, encoding an EGFR-family like glycoprotein [1]. Its amplification, which is strongly correlated with protein overexpression, occurs in about 15–43% of breast tumours [1-10].

The observation that morphologically similar neoplasmatic lesions of the breast can exhibit different biology has necessitated the identification of biological parameters that might improve risk assessment; the evaluation of HER-2/*neu *expression is a typical example [11]. Indeed, several studies have demonstrated that HER-2/*neu *amplification represents a prognostic and predictive marker; its expression is associated with early disease recurrence, relative resistance to chemo- and/or hormonotherapy and short survival [2,10]. In addition it has been shown that genetic alterations of the HER-2/*neu *oncogene represent early events involved in breast carcinogenesis and tumour initiation, while their presence is observed in all stages of malignant development from *in situ *carcinomas to metastatic lesions [12]. As a result, some researchers have maintained that HER-2/*neu *amplification and/or protein overexpression may also represent not only an important marker of prognosis but also a key indicator of the aetiological heterogeneity of breast carcinogenesis. [3,7-9].

On the other hand, the contribution of even well established breast cancer risk factors to the aetiology of carcinogenesis in the breast remains obscure, ill-defined and tenuous, mostly because of the existence of different pathways for the initiation and the evolution of a breast tumour [13]. In order to explain this incompatibility, several researchers have claimed that classification of tumours on the basis of HER-2/*neu *overexpression or amplification may define a subset of breast cancer in which the net effect of a risk factor could be rather more obvious and its impact on breast cancer development more clear [3,7,8].

Thus, a close correlation of a risk factor with HER-2/*neu *overexpression could indicate either that a HER-2/*neu *alteration is the way that this risk factor evolves into the carcinogenesis or that there is a parallel interaction between them that leads to breast tumour initiation and development. Since the data in the literature supporting the above hypothesis are few and conflicting, we decided to investigate, in a group of patients from a geographical area with a low incidence of breast cancer, whether HER-2/*neu *positive tumours are correlated with established or suspected risk factors for breast cancer and thus to identify distinct subgroups of high risk women.

## Methods

This study analysed data derived from the database of the Breast Unit of the Department of Surgical Oncology at the University General Hospital of Heraklion on the island of Crete, Greece. The study considered all women who were consecutively diagnosed with primary, invasive breast cancer in our unit from 1996 to 2002. Subjects of other races, ethnicity, with residence outside Crete or diagnosed with DCIS or LCIS were excluded. Finally, 384 women, all originating from the island of Crete, were eligible for analysis.

An age-stratified random sample of 566 women was used as a control group, derived from the Breast Unit's database of screened patients who had not developed breast cancer after a median follow up period of 40 months (range 12–92 months). Personal interviews were conducted with each woman during her first visit (both patients and controls) by a consultant or a senior resident. The interview followed a structured questionnaire, which did not change during the study period. Anthropometric measures were also made during the first visit.

Women were classified as postmenopausal if their menstrual cycles had ended naturally at least 12 months before the interview or from surgery or radiation therapy at any age. Those who reported not having menstrual cycles for the last 10 months were considered as perimenopausal and were combined with premenopausal women for the purpose of our analysis.

The following variables were analysed for all patients and controls: residence (rural/urban), age at interview (≤ 50 and >50 years), age at menarche (≤ 12 and >12 years old), age at first full birth (<23 vs. ≥ 23 years old), parity (nulliparous, 1 or 2, and >3), lactation (yes/no), use of medications to suppress lactation (yes/no), abortions and miscarriages (yes/no), age at menopause for postmenopausal women (≤ 50 and >50 years old), use of HRT for more than 2 months (yes/no), use of oral contraceptives for more than 2 months (yes/no), family history of breast cancer in a first degree relative (yes/no), history of benign breast disease (yes/no), obesity on the day of the interview (BMI ≤ 29 kg/m^2 ^vs. BMI>29 kg/m^2^, median value for the study population) and radiation history of the chest (yes/no).

### Immunohistochemical study

For this study, tumour blocks were successfully retrieved in 378 (98.4%) and in 377 (98.17%) of the 384 interviewed cases for the immunohistochemical detection of HER-2/*neu *protein and hormone-receptor expression, respectively.

### Immunohistochemical detection and scoring of the HER-2/neu protein expression

Immunohistochemistry with the monoclonal antibody CB11 (NCLCB11, Novocastra Laboratories, UK 12 8EW), at a dilution of 1/50 with incubation period of 60 min, was performed using the OPTIMAX automated system with the Super Sensitive Link-Label Detection System RTU Multilink AP/Fast Red, QA200OXE (purchased from Biogenex Laboratories, San Ramon CA 94583 USA), following antigen retrieval by microwave pre-treatment at 500 Watts for 3 × 5 min in citrate buffer (0.01 M, pH 6). Sections from breast cancer of known positivity were used as positive controls. Negative controls were processed by omitting the primary antibody and substituting non-immune serum. Scoring was based on the criteria recommended by DAKO A/S for the HercepTest [14]. Only membrane staining pattern and intensity were scored using the 0–3+ scale: scores of 0–1+ were considered negative, score 2+ was considered weak positive-need for FISH, and score 3+ was considered (strongly) positive.

### Immunohistochemical detection and scoring of estrogen and progesterone receptors

Three (3) μm-thick sections taken on negatively-charged (SuperFrostPlus) slides were dewaxed in xylene, and rehydrated through graded alcohols. Following antigen retrieval by microwave pre-treatment at 500 W for 3 × 5 min in citrate buffer (0.01 M, pH 6), estrogen receptor (ERa) and progesterone receptor (PR) expression was detected by immunohistochemistry using the same automated system and detection kit as above, and primary monoclonal antibodies to ERa (DAKO M7047) and PR (Biogenex code # MU 328-UC) at dilutions of 1/50 and 1/20, respectively, with incubation time 60', at room temperature. Positive and negative controls were processed as above. Positive nuclei counting was performed at a final magnification of 400× (Teaching double-headed NICON, ECLIPSE E400 microscope, equipped with CFI 10X/22 oculars). After scanning at a final magnification of 100× for locating the areas with highest density of ER+ or PR+ carcinoma cell nuclei (hot spots), a 40X/¥/0.17 WD 0.65 objective lens was used for cell counting. All carcinoma cells in three hot spots per immunostained slide were evaluated by two pathologists working simultaneously, though independently, and the mean of the two independent counts was considered the final counting value for each field and hot spot. The ratio of the ER+ or PR+ carcinoma cell nuclei was recorded separately for each of the hot spots. The final immunoreactivity index (score) was calculated as the mean percentage of ER+ or PR+ carcinoma cell nuclei in the three hot spots. Specimens were interpreted as positive for ER or PR if at least 10% of the cells demonstrated nuclear staining of any intensity of reactivity, from 1+ to 3+. Staining intensity was graded as negative (0), weak (1+), intermediate (2+) or strong (3+), and reported separately. A mean value of intensity was assigned for specimens in which the staining intensity varied from field to field, and/or from hot spot to hot spot.

### Statistical analysis

Odds ratios (OR) and 95% CI (confidence intervals), as approximators of relative risk, were calculated to measure the association of the groups of breast cancer and the risk factors, using the chi-square (χ^2^) test. A *p *value <0.05 was defined as significant. The potential association between breast cancers stratified by HER-2/*neu *status and well known predisposing factors was further investigated by using a stepwise logistic regression analysis (backward LR), testing the independent effect of breast cancer risk factors (independent variables) on breast cancer (dependent variable) for all women and also separately for premenopausal and postmenopausal females. In addition, we undertook further stratification with estrogen receptor status by using the same multivariate logistic regression model. These patient-controls odds ratios helped us to detect the pattern of heterogeneity and to explore plausible aetiological correlations between patient subgroups. Additionally, case-case odds ratios were calculated in order to measure the risk heterogeneity between HER-2/*neu*+ and HER-2/*neu*-tumours. It seems that the departure of the OR from unity can reveal the degree of heterogeneity between these subgroups [15].

## Results

Risk factor distributions in breast cancer patients and matched controls are presented in table 1. The mean age at interview was 56.30 years. When all patients were compared with matched controls, and after adjustment for confounding factors, an increased breast cancer risk was associated with only *four *of the factors examined: use of oral contraceptives (OR = 4.40, 95%C.I: 1.46–13.28), use of HRT (OR = 7.34, 95%C.I: 2.03–26.53), an age at first full pregnancy more than 23 years (OR = 1.91, 95%C.I: 1.29–2.83) and body mass index more than 29 kg/m^2 ^(OR = 3.13, 95%C.I: 2.02–4.84). Additionally, a history of abortion or miscarriage (OR = 0.56, 95%C.I: 0.38–0.82) was correlated with a decreased risk of breast cancer. However, the number of oral contraceptive and HRT users was too small for reliable estimates of risk.

**Table 1:** Characteristics of the participants

Factors	Cases N = 384 n(%)	Controls N = 566 n(%)	OR^1 ^(95% CI)	OR^2 ^(95% CI)
**Age at interview**				
≤ 50 years	138(36)	178(31)	1.00	
>50 years	246(64)	388(69)	0.82(0.62–1.08)	NS
**Area of residence**				
rural	189(49)	292(52)	1.00	
urban	195(51)	274(48)	0.91(0.70–1.18)	NS
**Menopausal status**				
Pre/perimenopausal	134(35)	170(30)	1.00	
Postmenopausal	250(65)	396(70)	0.80(0.60–1.06)	NS
**Age at menopause^3^**				
≤ 50 years	144(59)	252(64)	1.00	
>50 years	102(41)	140(36)	1.28(0.92–1.77)	NS
**Age of menarche**				
≤ 12 years	155(40)	150(27)	**1.86(1.41–2.45)**	
>12 years	229(60)	412(73)	1.00	NS
**Use of oral contraceptives**				
no	341(89)	548(97)	1.00	1.00
yes	43(11)	18(3)	**3.84(2.18–6.77)**	**4.40(1.46–13.28)**
**Use of HRT^3^**				
no	231(92)	393(99)	1.00	1.00
yes	19(8)	3(1)	**10.78(3.15–36.81)**	**7.34(2.03–26.53)**
**First degree family history**				
no	341(89)	522(92)	1.00	
yes	43(11)	44(8)	*1.5(0.96–2.33)*	NS
**Age at first full pregnancy**				
<23 years	106(35)	239(50)	1.00	1.00
≥ 23 years	197(65)	242(50)	**1.84(1.37–2.47)**	**1.91(1.29–2.83)**
**Parity**				
nulliparous	79(20)	78(14)	1.00	
1–2	175(46)	255(45)	**0.68(0.47–0.98)**	NS
3 plus	130(34)	233(41)	**0.55(0.38–0.80)**	NS
**Abortion or miscarriage**				
no	183(57)	247(50)	1.00	1.00
yes	138(43)	248(50)	**0.75(0.57–0.99)**	**0.56(0.38–0.82)**
**Lactation**				
no	67(22)	84(17)	1.00	
yes	238(78)	404(82)	0.74(0.52–1.06)	NS
**Medication to suppress lactation**				
no	273(90)	438(90)	1.00	
yes	31(10)	50(10)	0.99(0.62–1.60)	NS
**Radiation to the chest**				
no	372(97)	550(97)	1.00	
yes	12(3)	16(3)	1.11(0.52–2.37)	NS
**Body mass index**				
≤ 29 kg/m^2^	282(74)	498(88)	1.00	1.00
>29 kg/m^2^	97(26)	68(12)	**2.52(1.79–3.55)**	**3.13 (2.03–4.84)**
**Benign breast disease**				
no	315(82)	472(83)	1.00	
yes	69(18)	94(17)	1.10(0.78–1.55)	NS

^1^*Adjusted *for age. -^2^*Adjusted *for age, residence, menopausal status, menopausal age, menarche age, use of OC, use of HRT, first degree family history, age at first full pregnancy, parity, abortion, lactation, medication to suppress lactation, radiation to the chest, body mass index and benign breast disease. -^3^Postmenopausal women only.

**NS**: non significant multivariate OR. **Bold **types: statistically significant values.

Tumour characteristics of breast cancer patients are shown in table 2. Thirty eight percent (145/378) of the tumours showed HER-2/*neu *protein overexpression. HER-2/*neu *positive tumours were not related with menopausal state, age at interview, tumour size, grade and stage, nodal and estrogen receptor status, but there was a modest positive association between HER-2/*neu *and progesterone negative tumours.

**Table 2:** Characteristics of the tumours of breast cancer patients^1^.

Tumour characteristics	HER-2/*neu *+ n = 145 (%)	HER-2/*neu*- n = 233 (%)	*p *value
**Age at interview**			0.533
≤ 50 years	55 (40)	81 (60)	
>50 years	90 (37)	152 (63)	
**Staging**			0.106
I	30 (45)	36 (55)	
II	76 (35)	143 (65)	
III	20 (34)	39 (66)	
IV	2 (50)	2 (50)	
Unknown	17 (57)	13 (43)	
**Tumour size**			0.161
T1	55 (44)	71 (56)	
T2	65 (35)	121 (65)	
>T3	10 (29)	25 (71)	
Unknown	15 (48)	16 (52)	
**Menopausal status**			0.762
Pre/perimenopausal	52 (40)	80 (60)	
Postmenopausal	93 (38)	153 (62)	
**Grading**			0.577
I	7 (33)	14 (67)	
II	64 (36)	113 (64)	
III	60 (43)	80 (57)	
Unknown	14 (35)	26 (65)	
**Node Status**			0.119
Negative	55 (33)	112 (67)	
Positive	89 (43)	118 (57)	
Unknown	1 (25)	3 (75)	
Estrogen receptor status			0.108
Er+	60 (33)	120 (67)	
Er -	85 (43)	112 (57)	
Unknown		1	
**Progesterone receptor status**			**0.038**
Pr+	49 (49)	52 (51)	
Pr-	96 (35)	180 (65)	
Unknown		1	

*^1^Data for HER-2/neu status were missing for 6 of the 384 interviewed cases*.

### Menopausal status and estrogen receptor stratification

Subgroups of women stratified by menopausal status were further analysed by a multivariate stepwise logistic regression model adjusted for the remaining variables (table 3). In the *premenopausal *group of women, an increased risk for HER-2/*neu*-tumours was observed for those women who reported an age at first full pregnancy ≥ 23 years (*OR = 3.56, 95%C.I: 1.70–7.46*), a BMI>29 kg/m^2^(*OR = 6.89, 95%C.I: 2.23–21.25*), first degree family history (*OR = 3.30, 95%C.I:1.10–9.96*) or use of oral contraceptives (*OR = 11.19, 95%C.I 3.70–33.84*), while an age at menarche less than 12 years was the only factor which slightly increased the risk in premenopausal HER-2/neu+ patients (*OR = 2.09, 95%C.I 0.99–4.42*). Abortion played a less protective role (*p = 0.068*) for HER-2/*neu*-breast cancer in premenopausal than in postmenopausal women (*p = 0.038*). However, the *intercase *comparison in the premenopausal subgroup showed an evidence of heterogeneity only for the HER-2/*neu*+ women who had ever had an abortion (ratio of the *OR's = 3.12, 95%C.I:1.18–8.24*), while use of oral contraceptives (*OR = 0.16, 95%C.I: 0.04–0.60, p *= 0.007) and a positive first degree family history (*OR = 0.09, 95%C.I: 0.01–0.85, p *= 0.035) showed a stronger association for HER-2/*neu *negative tumours.

**Table 3:** Multivariate analysis of risk factors and HER-2/neu overexpression according to menopausal status

Risk Factors	HER-2/neu+ Cases/controls OR (95% CI)	HER-2/neu-Cases/controls OR (95% CI)	Ratio of the OR's Cases+/cases- OR (95% CI)
***PREMENOPAUSAL***			
Age at first full pregnancy(≥ 23 years)	NS	3.56(1.70–7.46)	NS
Body mass index(>29 kg/m^2^)	NS	6.89(2.23–21.25)	NS
Abortion or miscarriage(ever)	NS	0.49(0.23–1.05)	**3.12 (1.18–8.24)**
First degree family history(positive)	NS	3.30(1.10–9.96)	0.09 (0.01–0.85)
Use of oral contraceptives (ever)	NS	11.19(3.7–33.84)	0.16 (0.04–0.60)
Age of menarche (≤ 12 years)	2.09 (0.99–4.42)	NS	NS
***POSTMENOPAUSAL***			
Age at first full pregnancy(≥ 23 years)	2.19(1.23–3.91)	1.66(1.03–2.66)	NS
Body mass index(>29 kg/m^2^)	4.83(2.75–8.49)	2.67(1.56–4.55)	**2.23 (1.20–4.15)**
Abortion or miscarriage(ever)	0.50(0.28–0.88)	0.62(0.39–0.97)	NS
First degree family history(positive)	NS	2.23(1.08–4.63)	NS
Use of estrogens (ever)	NS	10.70(2.71–42.31)	0.21 (0.04–1.08)
Use of oral contraceptives (ever)	NS	6.47(1.89–22.16)	NS
Age of menarche (≤ 12 years)	NS	1.72(1.07–2.75)	0.54 (0.28–1.04)
***ALL WOMEN***			
Age at first full pregnancy(≥ 23 years)	2.19(1.23–3.91)	1.66 (1.03–2.66)	NS
Body mass index(>29 kg/m^2^)	4.83(2.75–8.49)	2.67(1.56–4.55)	**2.23(1.20–4.15)**
Abortion or miscarriage(ever)	0.50(0.28–0.88)	0.62(0.39–0.97)	NS
First degree family history(positive)	NS	2.23(1.08–4.63)	NS
Use of estrogens (ever)	NS	10.70(2.71–42.31)	0.21 (0.04–1.08)
Use of oral contraceptives (ever)	NS	6.47(1.88–22.16)	NS
Age of menarche (≤ 12 years)	NS	1.72(1.07–2.75)	0.54 (0.28–1.04)

*Adjusted *for age, residence, menopausal status, menopausal age, menarche age, use of OC, use of HRT, first degree family history, age at first full pregnancy, parity, abortion, lactation, medication to suppress lactation, radiation to the chest, body mass index and benign breast disease.

NS: non significant.

The results of logistic regression were identical for *all women *and the *postmenopausal *groups of patients due to the large sample size. Patients with an age of menarche ≤ 12 years (*OR = 1.72, 95%C.I: 1.07–2.75*), first degree family history (*OR = 2.23, 95%C.I:1.08–4.63*), use of HRT (*OR = 10.70, 95%C.I: 2.71–42.31*) or OC (*OR = 6.47, 95%C.I:1.89–22.16*) were at increased risk of developing HER-2/*neu*-breast cancer only, although the significance of the latter two factors was of little value due to the limited sample size. On the other hand, an age at first full pregnancy ≥ 23 years and a BMI greater than 29 kg/m^2 ^increased breast cancer risk independently of HER-2/*neu *status, while a history of abortion decreased risk in the same way. In *the case to case *comparison only BMI >29 kg/m^2 ^revealed a relative stronger connection with positive than with negative HER-2/*neu *tumours (ratio of *OR's = 2.23, 95%C.I: 1.20–4.15, p *= 0.011) and this may indicate an evidence of heterogeneity of a rather significant degree for this factor. The stronger association between an age at menarche ≤ 12 years, use of HRT and negative as opposed to positive HER-2/*neu *status did not reach statistical significance (*p *= 0.067 and p = 0.062, respectively).

A different stratification pattern of our study's population is presented in table 4. This multivariate model, further stratified by estrogen receptor status, confirmed the observed tight connections between HER-2/*neu *positivity and obesity already shown in the analysis so far. In more detail, although BMI >29 kg/m^2 ^elevated risk for both ER negative and positive tumours independently of HER-2/*neu status*, the association was significantly stronger for positive HER-2/*neu *tumours in the ER negative group (*ratio of OR's = 2.46, 95%CI: 0.97–6.21*). Additionally, in the same group an age at first full pregnancy >23 years revealed an increase of risk in both HER-2/*neu *groups, while first degree family history (*OR = 2.72, 95%C.I: 1.05–7.07, p *= 0.040), age at menopause >50 years (*OR = 2.05, 95%C.I: 1.10–3.79, p *= 0.023) and birth of 1–2 children (*OR = 2.38, 95%C.I: 1.21–4.67, p *= 0.012) elevated risk for HER-2/*neu *negative tumours only. In the ER+ group of women the direct comparison between cases revealed no associations with any factor at all, while abortion showed a protective pattern against breast cancer which expressed estrogen receptors independently of HER-2/*neu *status.

**Table 4:** Multivariate analysis of risk factors and HER-2/neu overexpression according to ER^1 ^status.

Risk Factors	HER-2/neu+ Cases/controls OR^2 ^(95% CI^3^)	HER-2/neu-Cases/controls OR (95% CI)	Ratio of the OR's Cases+/cases- OR (95% CI)
**ER + cases**			
Body mass index(>29 kg/m^2^)	5.59 (2.58–12.13)	2.84 (1.52–5.32)	NS
Age at 1st pregnancy (≥ 23 years)	2.09 (0.97–4.53)	NS	NS
First degree family history (positive)	NS	2.18 (0.92–5.14)	NS
Abortion or miscarriage (ever)	0.44 (0.20–0.95)	0.56 (0.32–0.99)	NS
**ER – cases**			
Body mass index(>29 kg/m^2^)	5.33 (2.59–10.94)	2.41 (1.15–5.04)	2.46 (0.97–6.21)
Age at 1st pregnancy (≥ 23 years)	2.37 (1.08–5.18)	1.78 (0.93–3.42)	NS
First degree family history (positive)	NS	2.72 (1.05–7.07)	NS
Age of menopause (>50 years)	NS	2.05 (1.10–3.79)	NS
Parity (1–2 children)	NS	2.38 (1.21–4.67)	NS

*Adjusted *for age, residence, menopausal status, menopausal age, menarche age, use of OC, use of HRT, first degree family history, age at first full pregnancy, parity, abortion, lactation, medication to suppress lactation, radiation to the chest, body mass index and benign breast disease.

^1^ER: estrogen receptor. NS: non significant.

## Discussion

This epidemiological study, conducted in a low incidence Mediterranean population, [16] found that obesity was related with postmenopausal breast tumours that overexpress HER-2/*neu *oncoprotein. In fact, increased BMI elevated risk in both groups, but the comparison between HER-2/*neu*+ and HER-2/*neu- *tumours revealed a much stronger association with HER-2/*neu*+ breast cancers.

Very few studies have examined the possibility whether HER-2/*neu *status can help discriminate aetiologically distinct subgroups of breast cancer cases, and none of them has identified the effect of increased BMI with HER-2/*neu *positive tumours [3,5,7-9].

More specifically, in contrast with other investigators who have shown an elevated risk for HER-2/*neu*+ tumours with an early age at first full pregnancy, we found a strong elevated risk with a late age regardless of HER-2/*neu *protein expression [3]. Previous findings suggested an inverse relationship between abortion and HER-2/*neu*+ breast cancers, while we also found this inverse association but independently of HER-2/*neu *status [7]. Interestingly enough, abortion increased risk for HER-2/*neu*+ tumours only in the premenopausal group of women. Early contraceptive use has been positively associated with HER-2/*neu*+ breast cancer in two studies [7,8], but our findings were different, revealing a positive association with HER-2/*neu *negative tumours. However, because the number of oral contraceptive (and HRT) users in this study was small, this subgroup analysis was hindered by decreased power to detect associations of any magnitude. The slightly protective effect of parity found in the age-adjusted analysis was diminished after logistic regression and did not reveal any association with HER-2/*neu *status, in contrast with previous findings [7]. Breastfeeding was associated with increased risks for breast cancer in women with HER-2/*neu *positive tumours in one study while other investigators reported opposite results [3,9]. Although our study population showed a remarkable lactation incidence (almost 80% of the participants) we found no associations at all.

Our findings have similarities and differences with respect to previous reports that examined the associations of breast risk factors with HER-2/*neu *status. This inconsistency may reflect differences in study design, populations, and laboratory methodology. In this study we used immunochemistry (CB11 monoclonal antibody) to assess the HER-2/*neu *protein overexpression, which is highly correlated with gene amplification according to previous reports [2,4]. Also, the percentage of women with breast cancer and HER-2/*neu *protein overexpression found here was within the limits reported elsewhere [3,5-9].

This lack of relationship between HER-2/*neu *protein overexpression and most of the hormone-related breast cancer risk factors does not completely agree with several hypotheses which have maintained that combined estrogen and HER-2/*neu *activation is closely involved in the same pathway in breast cancer carcinogenesis [17,18].

The only hormone-related factor that was found to be related with HER-2/*neu *positive tumours in our study was high body mass index, which is an established risk factor that has an estrogen-mediated oncogenic effect on the mammary gland. More specifically, obesity is associated with higher breast cancer risk among postmenopausal women through greater lifetime exposure to higher levels of estrogens produced in adipose tissue and lower SHBG production [19,20]. Higher levels of circulating estrogens enhance the rate of cell division, and this hormone-induced cellular proliferation can result in somatic mutations and finally lead to a malignant change. These alterations involve many genes, including those concerned with hormone metabolism and transport, DNA repair, as well as tumour suppressor genes and oncogenes such as the HER-2/*neu *gene [18,21]. According to some investigators, circulating estrogens can stimulate breast cancer cell proliferation, not only through hormone receptors, but also through the HER-2/*neu *receptor, and so promote carcinogenesis through common means [4,17,18].

Numerous epidemiological and experimental studies have shown the strong relationship between HER-2/*neu*-positivity and lack of hormone receptor expression in breast tumours [2,10,18,22]. In our study, HER-2/*neu *positive tumours were weakly related with the absence of estrogen receptors, although this was not statistically significant (see table 2). Because different ER status can result in different correlations between risk factors and HER-2/*neu+ *breast cancer, it is always important to examine these interactions under ER stratification [8]. Since antiestrogens can lower HER-2/*neu *levels in ER negative tumours, it is possible that an excess of estrogens can stimulate HER-2/*neu *in these tumours [8,18]. This mechanism could explain the stronger association between obesity (a situation with an overload of estrogens as mentioned above) and HER-2/*neu*-positivity among ER negative patients that was found in the present study (see table 5).

The interview was conducted during the subjects' first visit to the unit and before clinical examination or any other intervention took place. This constitutes an advantage, because there was no chance that the subjects (both cases and controls) would be influenced by the diagnosis and might therefore falsely inflate the relative risk. Thus, the likelihood of recall bias is not high, improving the comparability of several covariates in both groups, and the selection bias is lessened since all subjects had taken the same route through the Breast Unit's standard routine procedures.

Since each case group was compared with the same control group, any selection bias would be expected to have a similar effect on the estimates in the tumour subgroups. Thus, it is extremely unlikely that recall bias issues would apply only to those cases in a specific HER-2/*neu *status subgroup. Some caution regarding our findings is related to the size of the study group. In the analyses stratified by HER-2/*neu *and menopausal or ER status numbers are quite small and for some risk estimates the confidence intervals are wide and the estimates of risk unstable.

## Conclusions

In conclusion, our study did not confirm that the established or putative hormonal breast cancer risk factors differ regarding their relations with HER-2/*neu*+ versus HER-2/*neu*-breast tumours, with the exception of increased BMI. Further innovative studies with larger sample sizes are needed to examine how the status of these potentially modifiable breast cancer risk factors interacts with biological markers such as HER-2/*neu *oncoprotein. Their findings will provide us with greater insight into breast cancer aetiology and will help us identify any association that would help discriminate subgroups of women at higher risk.

## Abbreviations

EGFR: epidermal growth factor receptor, HRT: hormone

replacement therapy, BMI: body mass index, SHBG: sex hormone-binding protein, ER: estrogen receptor, PR: progesterone receptor, OC: oral contraceptives.

## Competing interests

The author(s) declare that they have no competing interests.

## Authors' contributions

**NT **conceived the study, participated in its design and drafted the manuscript.

**ES **participated in the design of the study, assisted in writing and reviewed the final article.

**EfS **and **KM **scheduled and performed the laboratory analysis.

**NA **performed the statistical analyses.

**VG **and **DDT **participated in the design and coordination of the study and reviewed the final article.

All authors have read, discussed and approved the manuscript.

## Pre-publication history

The pre-publication history for this paper can be accessed here:


